# Lepra in Italy

**Published:** 1844-06-01

**Authors:** 


### LEPRA IN ITALY.

At the scientific congress in Italy, Dr. Trompeo read a memoir on lepra, (the elephantiasis of the Greeks,) which he contended still existed in Europe, more especially in the Sardinian States; in the district of Nice, alone, he had seen a hundred lepers. He regarded it as not only hereditary, but contagious. Undeniable facts having convinced him that the disease is on the increase, he proposed that special hospitals should be opened for the reception of those afflicted with it, and that measures should be taken to separate them from the rest of the community. Dr. Costa confirmed the statements of Dr. Trompeo, and mentioned his having witnessed several instances of the transmission of lepra among the inhabitants of Ligaria. The congress offered a prize of the value of twelve pounds for the best memoir "On the Lepra of Italy, and on the means of preventing and curing that disease." The malady is a very different one from that which is called "lepra" in this country; it is also much more severe. It manifests itself under the form of numerous tubercles of variable size, livid, prominent, soft to the touch, which eventually suppurate, and occasion most hideous deformity. It is said by M. Roussel to exist endemically on the coast of the Mediterranean, and that in the Martigue Islands there are entire families of lepers.—*Med. Times, from London Lancet.*

